# Penile pseudomyogenic hemangioendothelioma/epithelioid sarcoma-like hemangioendothelioma: a case report and literature review

**DOI:** 10.3389/fonc.2026.1649382

**Published:** 2026-04-28

**Authors:** Wei Wang, Chang Liang, Haozhou Yang, Bin Wang, Jianghu Tang, Yubin Zhu, Ji Sun, Qingqiang Gao

**Affiliations:** 1 Department of Urology, Tianjin First Central Hospital, No.2 Baoshan West Road, Xiqing District, Tianjin, China; 2 Department of Urology, People's Hospital of Linshui, No. 487, Renmin North Road, Linshui County, Guangan, Sichuan, China; 3 Department of Urology, Fushun People's Hospital, No. 490, Jixiang Road, Fushun County, Zigong, Sichuan, China; 4 Department of Urology, Dianjiang Traditional Chinese Medicine Hospital, No. 502, Gongnong Road, Dianjiang County, Chongqing, China; 5 Department of Urology, People's Hospital of Luxian, No. 628 Longnao Avenue, Luxian, Luzhou, Sichuan, China; 6 Department of Andrology, No.2 People's Hospital of Fuyang, No. 1088, Yinghe West Road, Yingquan District, Fuyang, Anhui, China; 7 Department of Andrology, Nanjing Drum Tower Hospital, The Affiliated Hospital of Nanjing University Medical School, Nanjing, China

**Keywords:** case report, diagnosis, epithelioid sarcoma-like hemangioendothelioma, histopathology, immunohistochemistry, penile nodule, pseudomyogenic hemangioendothelioma

## Abstract

Pseudomyogenic hemangioendothelioma (PHE) is a rare vascular neoplasm predominantly affecting young adult males, often presenting in soft tissues of the extremities, with exceptionally rare cases in the penile region. This report details the case of a 45-year-old Han Chinese male with a painful penile nodule misdiagnosed as Peyronie’s disease due to its clinical presentation. Initial evaluations, including ultrasound and laboratory tests, suggested uncertainty regarding the lesion’s nature. Surgical excision was performed, revealing a dark purplish-red mass embedded in the tunica albuginea. Histopathological analysis supported a diagnosis of PHE, highlighted by immunohistochemical markers including strong positivity for FOSB and CD31. The postoperative course demonstrated good recovery and resolution of symptoms with no recurrence observed during follow-up. This case emphasizes the diagnostic challenge posed by PHE due to its deceptive clinical features and the necessity for histopathological examination coupled with immunophenotypic profiling for accurate diagnosis. Increased awareness of PHE is crucial for urologists and andrologists to improve diagnostic accuracy and facilitate timely management of this rare entity.

## Introduction

Pseudomyogenic hemangioendothelioma (PHE) is a rare vascular tumor that predominantly occurs in young adults, with a higher incidence observed in male patients. This tumor typically arises in the superficial or deep soft tissues of the extremities; however, there are also rare cases involving the bones or visceral organs. Even less common are tumors located in the penile region (1, 2). To date, only seven cases of adult penile pseudomyogenic hemangioendothelioma with detailed clinical information have been reported (**Table 1**).

**Table 1:** Clinical data from 8 patients.

Case	Age	Time of onset	Site	Pain	Solitary/Multifocal	Treatment	Followup	Recurrence	ED	Reference
1	30	2M	G	No	Solitary	No	NA	No	NO	(1)
2	43	6M	P/G	Yes	Multifocal	LE	9M	Yes	NA	(12)
3	18	NA	P/S	NA	Multifocal	LE	26M	No	NA	(6)
0 4	27	NA	P	NA	Solitary	NA	9M	Yes	NA	(6)
5	37	3M	G	Yes	Multifocal	LE	12M	No	No	(7)
6	19	12M	P	Yes	Solitary	LE+RCS	6M	No	No	(13)
7	47	NA	G	Yes	Solitary	LE	2M	Yes	NA	(2)
8	45	2W	P	Yes	Solitary	LE	9M	No	No	ourcase

M, Month; W, Week; G, Glan penis; P, Penile shaft; S, Scrotum; NA, not applicable; LE, bcalexcision; RCS, reconstruction.

Due to the rarity of such cases, along with clinical features that bear some resemblance to Peyronie’s disease, it can be challenging for urologists or andrologists to arrive at an accurate diagnosis when encountering this tumor. Moreover, from a histological perspective, its epithelial-like morphology and the absence of significant vascular differentiation on hematoxylin and eosin (HE) staining can easily lead to misinterpretation as other lesions, resulting in varied treatment approaches. Therefore, we present a case of pseudomyogenic hemangioendothelioma, detailing its diagnostic and therapeutic processes as well as pathological findings. In addition, we review the characteristics of previously reported cases of penile pseudomyogenic hemangioendothelioma to enhance awareness of this condition among relevant medical professionals.

## Case presentation

**Figure 1:**
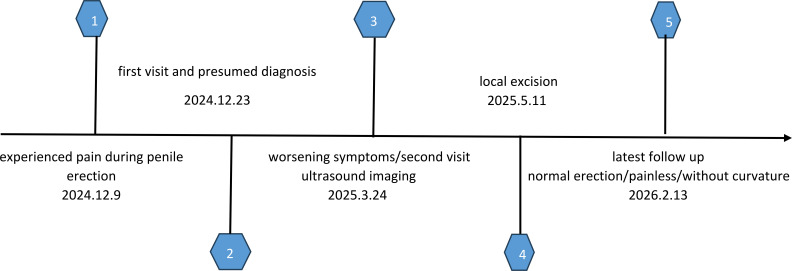
Summary timeline of care process.

A 45-year-old Han Chinese male presented to the outpatient clinic on December 23, 2024 (**Figure 1**), with a painful penile nodule. Two weeks prior, he had experienced pain during penile erection and noticed a small, tender subcutaneous nodule at the base of his penis.

Upon initial examination, no erythema, swelling, or visible protrusion was observed on the dorsal aspect of the penile base. Palpation revealed a 0.5 cm, tender nodule located on the tunica albuginea. Given the small size of the lesion, Peyronie’s disease could not be ruled out. the patient reported increased pain that impacted his daily activities. On re-examination, a subcutaneous nodule, approximately 5 mm in diameter, was noted at the dorsal base of the penis. It was tightly adherent to the tunica albuginea, immobile, and markedly tender, with no involvement of the overlying skin. Ultrasound imaging revealed a hypoechoic nodule in the dorsal subcutaneous region, measuring 6 x4 mm, connected to the tunica albuginea, with heterogeneous internal echogenicity. No significant blood flow was detected within or around the lesion.

The patient had no significant comorbidities. He had no history of hypertension, diabetes, dyslipidemia, or cerebrovascular disease. He also denied symptoms of lower urinary tract involvement, such as abnormal urinary frequency, painful micturition, or hematuria. His medical history was negative for substance use (tobacco-native, alcohol-abstinent) and familial cancer predisposition.

At the time of examination, his vital signs were stable: body temperature of 36.8 °C, heart rate of 72 bpm, blood pressure 116/68 mmHg, and respiratory rate of 16 breaths per minute. Abdominal examination revealed no palpable masses or visceromegaly. Neurological examination was unremarkable, showing intact cranial nerve function and normal sensorimotor responses.

Laboratory tests, including complete blood count, liver and renal function tests, and endocrine evaluation (total testosterone: 15.2 nmol/L), were within normal limits. Tumor markers (AFP3.1 ng/mL, CEA 2.4 μg/L, CA-125 12 U/mL) were also within normal range. Electrocardiography showed regular sinus rhythm without ischemic changes.

Given the uncertain nature of the lesion and increasing pain, surgical excision was recommended for definitive diagnosis. The patient was admitted and underwent surgical resection under general anesthesia on May 11, 2025.

**Figure 2:**
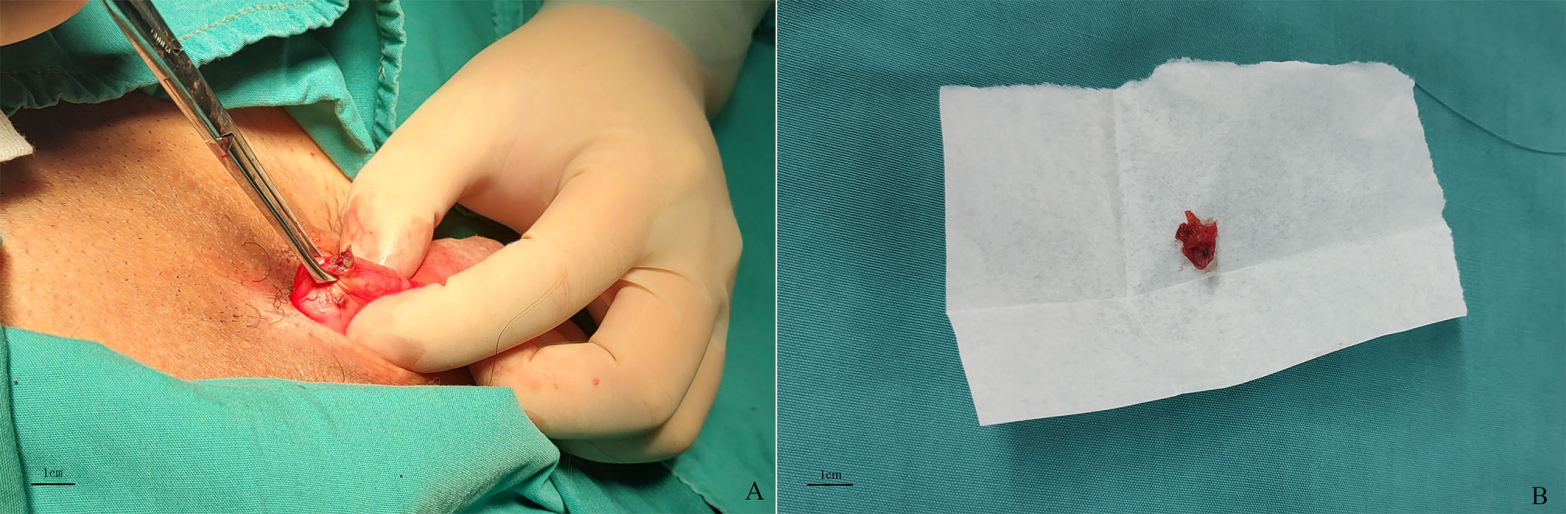
Lesions of the tunica albuginea at the base of the penis and the gross appearance of the tumor. **(A)**: Lesions have a dark purplish-red to brownish-yellow appearance on the epidermis. **(B)**: Size of the excised tissue is about 12x8 mm.

During the surgery, penile degloving was performed to expose Buck’s fascia at the base. The fascia appeared intact, and after opening Buck’s fascia, a dark purplish-red mass embedded in the tunica albuginea was identified. The mass had indistinct borders and was grasped with an Allis clamp. A longitudinal spindle-shaped excision of the lesion, with a 2mm margin, was performed (**Figure 2A**). The tunica albuginea and Buck’s fascia were subsequently repaired.

**Figure 3:**
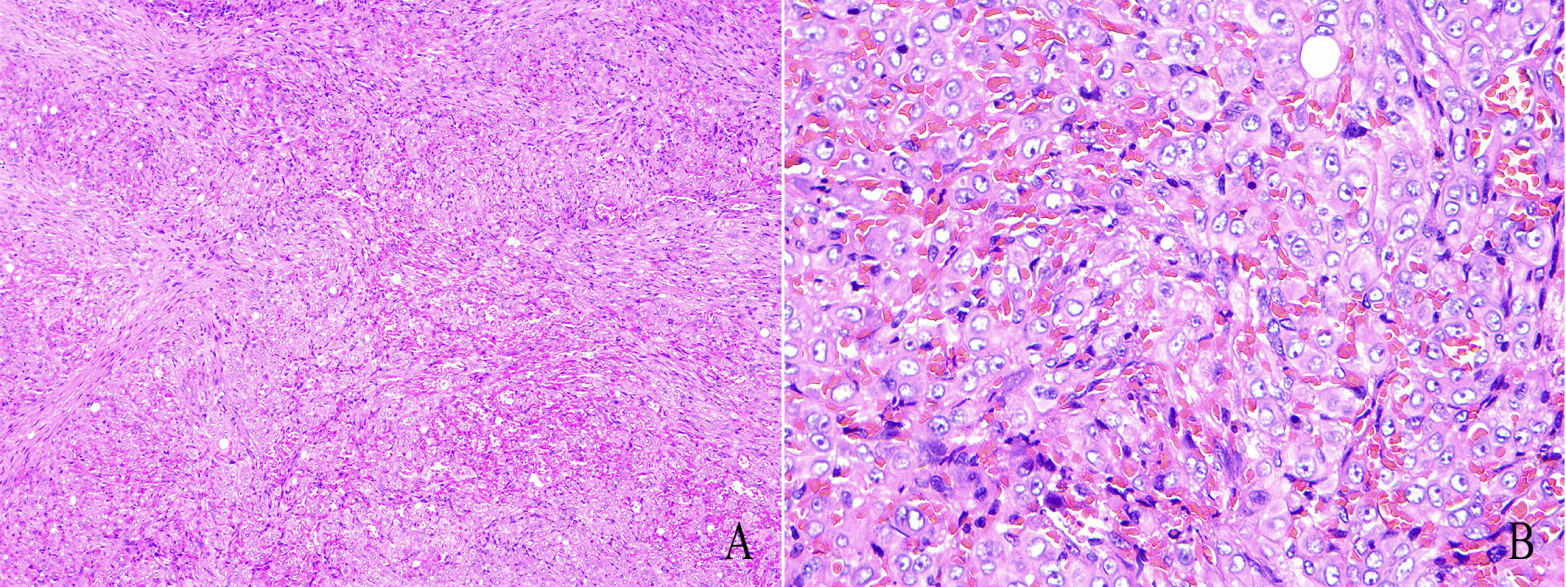
Results of histopathology. **(A)**: The tumor is composed of spindle cells and poorly differentiated vasculature, with a disorganized arrangement of the spindle cells. (HE, 10×). **(B)**: The nuclei are spindle-shaped or oval, with visible nucleoli. Mitotic figures are not prominent, mild nuclear atypia is observed, and the cytoplasm is unclear. (HE, 40×).

The excised specimen measured 12x8mm and exhibited a dark purplish-red to brownish-yellow appearance (**Figure 2B**). Histopathological examination revealed that the exact nature of the mass remained undetermined, but pseudomyogenic hemangioendothelioma (PHE) could not be excluded (**Figures 3A, B**).

**Figure 4:**
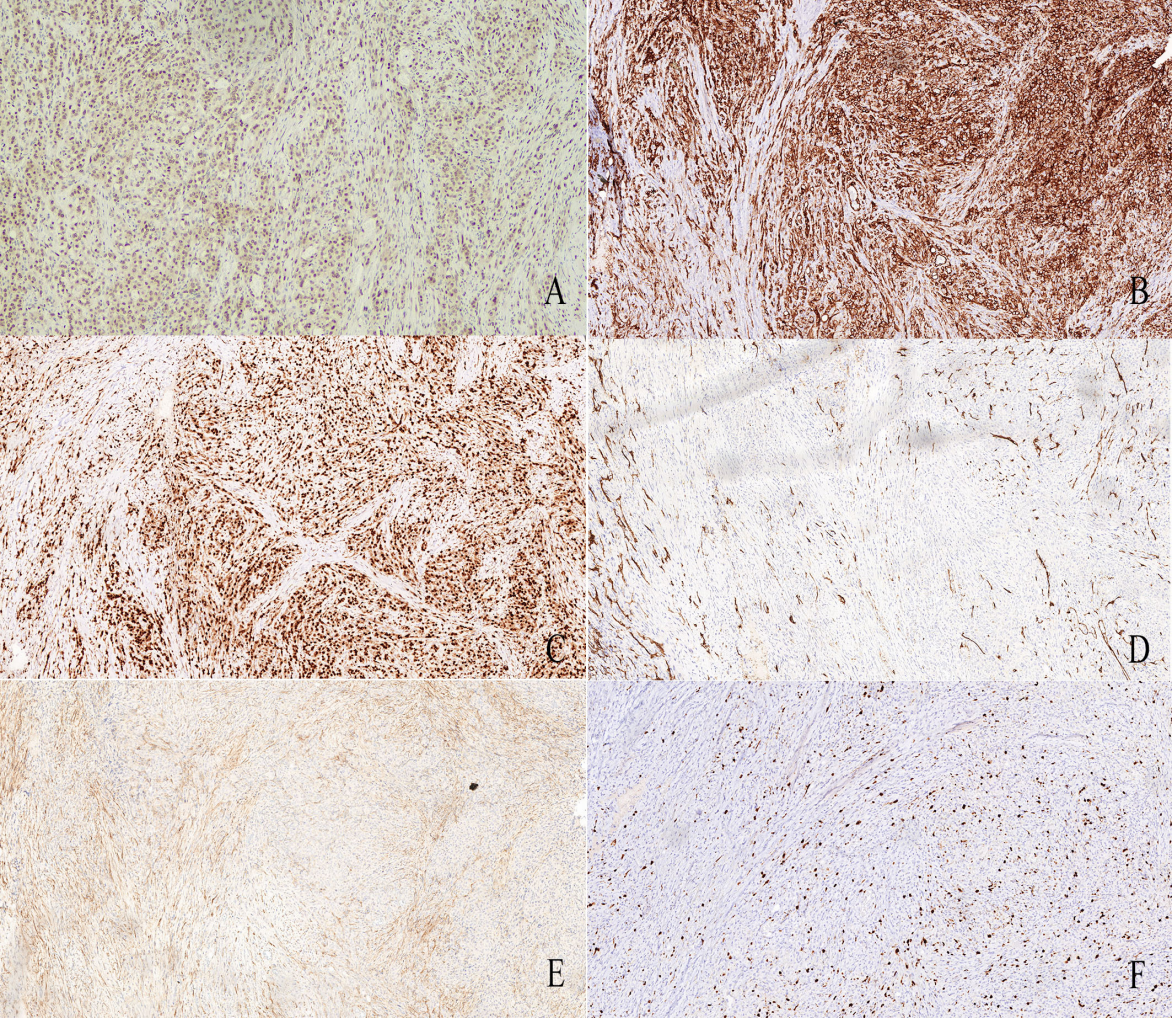
Typical results of immunohistochemistry. **(A)**: Strong positive expression of FOSB(+++) in the tumor (10×); **(B)**: Strong positive expression of CD31 in the tumor (10×); **(C)**: Strong positive expression of ERG in the tumor (10×); **(D)**: Negative expression of CD34 in the tumor (10×); **(E)**: Negative positive expression of EMA in the tumor (10×); **(F)**: Expression of 10% Ki-67 index (10×).

Immunohistochemical analysis demonstrated strong positivity for FOSB (+++) (**Figure 4A**), CD31 (+++) (**Figure 4B**), moderate positivity for ERG (++) (**Figure 4C**), and negativity for CD34 (**Figure 4D**) and EMA (**Figure 4E**). The Ki-67 index was approximately 10% (**Figure 4F**), which supported the diagnosis of pseudomyogenic hemangioendothelioma.

Following a multidisciplinary team discussion, postoperative surveillance with regular follow-up was recommended. The first follow-up, two weeks post-surgery, revealed good wound healing and resolution of penile pain. At six weeks, the patient reported normal erectile function with no pain or curvature. At ten weeks, he remained asymptomatic with no evidence of recurrence.

Subsequent follow-up appointments were scheduled every three months. The patient was instructed to seek medical attention immediately should the disease progress. At the most recent follow-up on February13, 2026, there was no evidence of recurrence, and erectile function remained normal, with no pain or curvature.

## Discussion

Pseudomyogenic hemangioendothelioma (PHE) is a rare vascular neoplasm with intermediate malignant potential. It was first described in 1992 by Mirra et al. (3)as a fibroma-like variant of epithelioid sarcoma, reflecting its spindle cell morphology and overlapping features with fibrohistiocytic and myogenic tumors. In 2003, Billings etal. (4) reported a series of seven similar cases and proposed the term “epithelioid sarcoma-like hemangioendothelioma”, recognizing its endothelial immunophenotype despite the absence of well-formed vascular differentiation. Subsequently, Hornick et al. analyzed a larger cohort and confirmed its vascular origin and relatively indolent biologic behavior, leading to the adoption of the current term pseudomyogenic hemangioendothelioma. In 2013, the World Health Organization classified PHE as a rare vascular tumor with intermediate malignant potential and infrequent metastasis (5).

PHE predominantly affects young adult males and most commonly arises in the extremities, particularly the lower limbs. Lesions are frequently multifocal within a single anatomic region and may involve multiple tissue planes, including dermis, subcutis, skeletal muscle, and sometimes bone (6). Osseous involvement may be associated with bone destruction and pain. Primary involvement of the penis is exceptionally rare. To date, only 11 cases have been reported in the literature, of which 7 provide relatively detailed clinical information (**Table 1**).

Clinically, PHE exhibits heterogeneous manifestations and a tendency for local recurrence, whereas distant metastasis is uncommon. Progressive pain is considered a characteristic feature in many patients, although not universally present (7). In the present case, the patient presented with a painful nodule at the penile root, clinically resembling Peyronie’s disease. Peyronie’s disease typically manifests as a palpable plaque or nodule within the tunica albuginea, often associated with erectile pain, particularly during the inflammatory phase. In our patient, the lesion was small, firm, embedded within the tunica albuginea, immobile on palpation, and associated with progressively worsening tenderness—features that closely mimicked Peyronie’s disease. The absence of ultrasonographic evaluation, together with the extreme rarity of penile PHE and cognitive diagnostic bias, contributed to the initial misdiagnosis. However, the persistence and progression of pain during observation prompted reconsideration of the diagnosis. Further imaging and surgical excision with histopathological evaluation ultimately established the correct diagnosis of PHE.

This case emphasizes that progressive painful penile nodules without a clear history of trauma should raise suspicion for uncommon vascular tumors, including PHE, particularly when the clinical course is atypical. Nevertheless, clinical findings alone are insufficient for diagnosis, and histopathological examination remains essential.

## Histopathologic and immunophenotypic features

Histologically, PHE is characterized by infiltrative growth of loosely arranged fascicles or sheets of epithelioid to spindle-shaped cells with abundant eosinophilic cytoplasm, vesicular nuclei, and small but distinct nucleoli. Nuclear atypia is generally mild, and mitotic activity is low (5). Necrosis is uncommon. Immunohistochemically, PHE typically demonstrates diffuse expression of cytokeratin AE1/AE3 and endothelial-associated transcription factors such as FLI1 and ERG. Approximately half of cases express CD31, while CD34 is usually negative (8). Focal smooth muscle actin expression may be observed in a subset of tumors. Importantly, diffuse nuclear immunoreactivity for FOSB has been identified in the vast majority of cases and represents a highly sensitive diagnostic marker (9). In contrast, most histologic mimics are negative for FOSB, with rare exceptions in isolated cases of epithelioid hemangioendothelioma or angiosarcoma. INI1 expression is retained, which helps distinguish PHE from epithelioid sarcoma (8).

At the molecular level, PHE frequently harbors a t(7;19)(q22;q13) translocation resulting in a SERPINE1-FOSB gene fusion. The SERPINE1 promoter likely drives overexpression of FOSB (9, 10). Although FOSB rearrangements may also be detected in a subset of epithelioid hemangiomas, SERPINE1-FOSB fusion appears characteristic of PHE. Additional FOSB rearrangements without SERPINE1 involvement have also been described (11, 12). Detection of FOSB gene rearrangement can therefore serve as a useful adjunct in challenging diagnostic cases.

## Differential diagnosis

Primary epithelioid vascular tumors of the penis are exceedingly rare, and significant morphologic and immunophenotypic overlap exists among entities such as epithelioid hemangioendothelioma (EHE), epithelioid angiosarcoma (EAS), and epithelioid sarcoma (ES).

Compared with EHE, which typically exhibits cords or nests of epithelioid endothelial cells embedded within a characteristic myxohyaline stroma and often shows intracytoplasmic vacuoles(“blister cells”), our case lacked both the typical myxohyaline matrix and well-formed intracytoplasmic lumina. Furthermore, CD34 expression—commonly positive in EHE—was completely negative in this tumor. Diffuse nuclear FOSB positivity further argues against conventional EHE.

Epithelioid angiosarcoma is a high-grade malignant vascular tumor characterized by marked cytologic atypia, frequent mitoses, necrosis, and destructive infiltrative growth. Although EAS may express CD31 and ERG, it is typically associated with a markedly elevated proliferative index. In contrast, our case demonstrated only mild-to-moderate nuclear atypia, absence of extensive necrosis, lack of complex anastomosing vascular channels, and a Ki-67 index of approximately 10%, supporting exclusion of high-grade angiosarcoma.

Epithelioid sarcoma may present as nodular lesions in young patients and can mimic PHE morphologically. However, ES typically shows central necrosis and diffuse expression of epithelial markers such as EMA, and lacks definitive endothelial markers. In the present case, EMA was negative, CD31 and ERG positivity supported endothelial differentiation. Diffuse nuclear FOSB expression further excluded epithelioid sarcoma.

Taken together, the absence of myxohyaline stroma and intracytoplasmic lumina, lack of high-grade cytologic features, negativity for epithelial markers, CD34 negativity, diffuse nuclear FOSB positivity, and a relatively low proliferative index strongly support the diagnosis of PHE.

## Management and prognosis

Complete surgical excision with histologically negative margins remains the cornerstone of treatment for PHE. Although PHE is classified as a tumor with intermediate biologic behavior and a recognized propensity for local recurrence, distant metastasis is uncommon. In the limited number of reported penile PHE cases (**Table 1**), local recurrence has been described in a subset of patients (13), underscoring the need for careful postoperative surveillance. In the present case, the definitive diagnosis was established based on postoperative histopathological examination. The lesion was small and completely excised with negative margins, without high-risk pathological features suggestive of aggressive behavior. Given these favorable findings, the focus shifted from immediate oncologic control to individualized postoperative management.

Preservation of erectile function and overall quality of life were important considerations in this anatomically and functionally sensitive location. After discussing the pathology results with the patient, we engaged in a thorough conversation about the implications of the diagnosis and the need for postoperative surveillance. The patient was made aware of the risks of local recurrence, and we discussed the uncertain benefits of additional interventions beyond careful monitoring. It was emphasized that close follow-up could effectively mitigate risks while respecting the patient’s desire to avoid overtreatment. This shared decision-making process reinforced the importance of patient-centered care, allowing the patient to express his preferences and concerns. No evidence of recurrence has been observed during 9 months of follow-up, and overall, the patient reported satisfaction with the diagnostic process, surgical outcome, and follow-up approach.

## Contribution to current knowledge

This case report presents a rare instance of primary penile PHE, offering new insights into the clinical characteristics and management of this uncommon tumor. Notably, this case uniquely illustrates the potential for confusion between PHE and Peyronie’s disease, as evidenced by the patient’s symptoms—painful nodules at the penile base resembling those seen in Peyronie’s disease. This underscores the complexities inherent in the overall diagnostic process and emphasizes the need for vigilance regarding rare vascular tumors. Through detailed immunohistochemical analysis, specifically highlighting the diffuse nuclear expression of FOSB, combined with clinical presentation and histopathological features, we further reinforced the diagnosis of penile PHE, emphasizing the necessity of considering PHE in differential diagnoses of tumors affecting the penis and external genitalia.

## Expanded management options and outcomes

Previous literature on cases of PHE of the penis and external genitalia has primarily emphasized local surgical excision to achieve negative margins. Our case not only adhered to these treatment protocols but also prioritized the preservation of erectile function and quality of life, selecting a monitoring strategy for postoperative management to avoid unnecessary overtreatment. This report summarizes the clinical management of this rare penile PHE, emphasizing the significance of individualized treatment plans that ensure oncologic safety while preserving patient functionality and quality of life. The synthesis of different management approaches and outcomes from extant literature can inform clinical decision-making for future cases of penile PHE.

## Limitations

Despite the valuable contributions of this case report, it is essential to acknowledge several limitations. As a single case study, the generalizability of our findings is limited. The short follow-up duration and the absence of molecular confirmation and long-term functional data restrict the depth of our analysis. Additionally, the decision not to conduct molecular testing for FOSB-related fusion genes in this instance somewhat constrains the diagnostic rigor of our report. Future studies should prioritize comprehensive molecular testing, especially in atypical cases with unusual clinical presentations and immunophenotypes, to strengthen diagnostic validation.

## Data Availability

The original contributions presented in the study are included in the article/supplementary material. Further inquiries can be directed to the corresponding author.
